# Raltegravir plus abacavir/lamivudine in virologically suppressed HIV-1-infected patients: 48-week results of the KIRAL study

**DOI:** 10.1371/journal.pone.0198768

**Published:** 2018-06-14

**Authors:** Jesús Troya, Rocio Montejano, Pablo Ryan, Cristina Gómez, Mariano Matarranz, Alfonso Cabello, Francisco Vera, María Antonia Sepúlveda, Ignacio Santos, Gloria Samperiz, Pablo Bachiller, Vicente Boix, Pilar Barrufet, Miguel Cervero, José Sanz, Javier Solís, María Yllescas, Eulalia Valencia, on behalf of the GESIDA-8715 Study Group

**Affiliations:** 1 Hospital Universitario Infanta Leonor, Madrid, Spain; 2 Hospital Universitario La Paz, Madrid, Spain; 3 Hospital Universitario Ramón y Cajal, Madrid, Spain; 4 Hospital Universitario 12 de Octubre, Madrid, Spain; 5 Hospital Universitario Fundación Jiménez Díaz, Madrid, Spain; 6 Hospital General Universitario Santa Lucia, Murcia, Spain; 7 Hospital Virgen de la Salud, Toledo, Spain; 8 Hospital Universitario La Princesa, Madrid, Spain; 9 Hospital Universitario Miguel Servet, Zaragoza, Spain; 10 Hospital Universitario Río Hortega, Valladolid, Spain; 11 Hospital General Universitario de Alicante, Alicante, Spain; 12 Hospital de Mataró, Mataró, Spain; 13 Hospital Universitario Severo Ochoa, Madrid, Spain; 14 Hospital Universitario Príncipe de Asturias, Madrid, Spain; 15 Fundación SEIMC-GESIDA, Madrid, Spain; Azienda Ospedaliera Universitaria di Perugia, ITALY

## Abstract

**Background:**

Long-term combination antiretroviral therapy often results in toxicity/tolerability problems, which are one of the main reasons for switching treatment. Despite the favorable profile of raltegravir (RAL), data on its combination with abacavir/lamivudine (ABC/3TC) are scarce. Based on clinical data, we evaluated this regimen as a switching strategy.

**Design:**

Multicenter, non-controlled, retrospective study including all virologically suppressed HIV-1-infected patients who had switched to RAL+ABC/3TC.

**Methods:**

We evaluated effectiveness (defined as maintenance of HIV-1-RNA <50 copies/mL at 48 weeks) safety, tolerability, laboratory data, and CD4+ count at week 48 of this switching strategy.

**Results:**

The study population comprised 467 patients. Median age was 49 years (IQR: 45–53). Males accounted for 75.4%. Median CD4+ count at baseline was 580 cells/μL (IQR, 409). The main reasons for switching were toxicity/tolerability problems (197; 42.2%) and physician’s criteria (133; 28.5%). At week 48, HIV-1 RNA remained at <50 copies/mL in 371/380 (97.6%; 95%CI: 96.4–99.0) when non-virological failure was censured. Virological failure was recorded in 1.9% patients and treatment failure in 20.5% of patients (96/467 [95%CI, 16.9–24.2]). The main reasons for treatment failure included switch to fixed-dose combination regimens (31; 6.6%), toxicity/poor tolerability (27; 5.8%), and physician’s decision (17; 3.6%). A total of 73 adverse events were detected in 64 patients (13.7%). These resolved in 43 patients (67.2%). Of the 33 cases related or likely related to treatment, 30 were Grade-1 (90.9%). CD4+ count and renal, hepatic, and lipid profiles remained clinically stable over the 48 weeks.

**Conclusions:**

Our findings suggest that RAL+ABC/3TC could be an effective, safe/tolerable, and low-toxicity option for virologically suppressed HIV-1-infected patients.

## Introduction

The availability of highly effective combination antiretroviral therapy (cART) over the last 2 decades has made HIV-1 infection a treatable chronic disease. However, the need for continued use of cART has generated problems such as toxicity, lack of tolerability, and drug interactions, which have forced clinicians to seek new treatment regimens. These regimens are not always adequately represented in clinical trials or observational studies [1,2].

Toxicity has been a major concern for clinicians engaged in managing HIV-1 infection, and despite the good safety and tolerability profiles of new drugs, it remains a problem in some patients. [3]. In many cases, cART-induced toxicity has been attributed to tenofovir disoproxil fumarate (TDF) [4–6], which, when combined with emtricitabine (FTC), has been included in most regimens as a recommended agent for naïve patients in international guidelines [7, 8]. The alternative backbone with abacavir plus lamivudine (ABC/3TC) is widely used in clinical trials and also in real-world practice, especially when clinicians wish to avoid the toxicity associated with TDF [9, 10].

Raltegravir (RAL) is an integrase strand transfer inhibitor (INSTI) with a very good safety/tolerability profile. It was approved on July 8, 2009 by the US Food and Drug Administration for the treatment of naïve HIV-1-infected patients in combination with 2 nucleoside analogues [11]. Abundant data from clinical trials and some real-life cohort studies in naïve patients indicate that the combination of RAL with FTC/TDF, and to a lesser extent with ABC/3TC, is highly efficacious and safe [12–14]. As a switching option, RAL has been studied mostly in combination with FTC/TDF [15, 16], and available data associated with ABC/3TC are scarce [17, 18], even though this regimen has been widely used in clinical practice.

Therefore, the objective of the present study was to determine the effectiveness and safety of RAL plus ABC/3TC as a switching strategy in virologically suppressed HIV-1-infected patients based on real-life data.

## Methods

### Study design and patients

We performed a multicenter, non-controlled, retrospective study of virologically suppressed HIV-1-infected patients switching to RAL (400 mg bid or 800 mg qd) plus ABC/3TC in 14 hospitals across Spain. All patients included fulfilled the following criteria: i) age ≥18 years, ii) documented HIV-1 infection, iii) switch from another regimen to RAL+ABC/3TC for any reason, iv) serum HIV-1 RNA <50 copies/mL for at least 24 weeks before switching to RAL+ABC/3TC, and v) availability of HIV-1 viral load records during follow-up including baseline, intermediate, and week-48 values when appropriate. The study data were collected between January and February 2017 from patients who had switched from December 2007 to January 2016 to ensure that at least 48 weeks of clinical follow-up were available. Eligible individuals were identified through the records obtained from clinical databases and/or from the hospital pharmacy unit.

Data for the study were collected retrospectively from the patient’s medical records, anonymized, and entered into an on-line electronic database (REDCap, Research Electronic Data Capture) [19]. Baseline demographic and HIV-related data were collected. Laboratory results (blood count, biochemical parameters including lipid profile and hepatic and renal function, CD4+ lymphocyte count, and HIV-1 RNA) and adverse events (AEs) were recorded at baseline and at the follow-up visits (every 12 to 24 weeks), depending on the routine clinical protocols at each hospital. The reasons for treatment discontinuation in patients who stopped or switched therapy and the results of genotypic resistance testing after virological failure were included when available.

The study protocol was approved by Hospital Universitario Gregorio Marañón Ethics Committee (code LEO-RAL-2015-01) in accordance with the principles of the Declaration of Helsinki (2013). The study was also approved by the individual ethics committees in some centers (H. Ramón y Cajal, Fundación Jiménez-Díaz, H. Virgen de la Salud, H 12 de OCtubre, H. Miguel Servet, H. General de Alicante, H. Santa Lucia, H. Mataró, H. Río Ortega, H. La Princesa, H. Severo Ochoa, H. Príncipe de Asturias). The data analysis was based on retrospective and anonymized routine clinical data, thus obviating the need for written informed consent.

### Study endpoints

#### Primary endpoint

The primary endpoint was the percentage of patients who maintained virological suppression (HIV-1 RNA <50 copies/mL) after 48 weeks of treatment. Virological failure was defined as 2 consecutive HIV-1 RNA measurements >50 copies/mL or a single measurement of >50 copies/mL if treatment was subsequently changed. Viral load was evaluated 3 or 4 times, when available, during the 48-week period of the study to ascertain virological control, according to the routine clinical protocols at each hospital. If viral load was not available in the records at week 48, or in the following 4–8 weeks, patients were considered treatment failures.

#### Secondary endpoints

The secondary endpoints included the proportion of patients with treatment failure due to any cause, clinical/laboratory AEs, description and severity of AEs, and changes from baseline in the following: a) serum total cholesterol, LDL-cholesterol, HDL-cholesterol, and triglycerides; b) serum aspartate aminotransferase (AST), alanine aminotransferase (ALT), and alkaline phosphatase (AP) levels; and c) estimated glomerular filtration rate (eGFR) according to the CKD-EPI equation.

Treatment was considered to have failed when any of the following events occurred: 1) virological failure; 2) interruption of treatment by the patient or the physician; 3) change of treatment regimen for reasons not related to virological failure including simplifications or AEs; and 4) incomplete data or missing patients.

We also conducted a survival analysis, in which patient data were censored if the cause of treatment failure was not virological failure or treatment toxicity.

### Statistical analysis

Nominal variables were described as numbers and percentages. Variables that did not follow a normal distribution were described using the median and interquartile range (IQR: 25–75). The association between qualitative variables was assessed using the χ^2^ test when the sample was sufficiently large or Fisher’s exact test when it was not; a Yates correction was used when necessary.

The percentage of patients who maintained virological suppression was analyzed using Kaplan-Meier curves, and including all treatment failures. The 95%CI for the proportion of patients at risk of treatment failure at 48 weeks was derived from the Kaplan-Meier curve.

Continuous variables were homoscedastic and were normally distributed according to the Levene and Kolmogorov-Smirnov tests. The values of each continuous variable at the different time points were considered dependent measures. Thus, changes in these variables over the 48 weeks of treatment were analyzed using mixed linear models (MLM) with an autoregressive covariance structure, and results were confirmed by general linear models (GLM) with repeated measures. The significance of differences time points for each continuous variable was determined by least significant difference analyses.

All analyses were performed using R v.3.3.2 (R Development Core Team 2014) [20].

## Results

### Characteristics of the patients

The study population comprised 467 patients. The baseline characteristics of the study population are summarized in Table 1. Men accounted for 75.4% of the population, and the median age was 49 years (IQR: 45–53). The most frequent HIV-1 risk factor in the study population was intravenous drug use (226, 48.4%). Approximately half of the patients (228, 48.8%) had prior AIDS-defining conditions, and a third (156, 33.4%) had active hepatitis C virus (HCV) co-infection. At baseline, the median CD4+ count was 580 cells/μL (IQR: 372–781), and the median CD4+ nadir was 169 cells/μL (IQR: 65–274). Median time since HIV-1 diagnosis before switching to RAL+ABC/3TC was 17 years (IQR: 10–22), and median time with undetectable HIV-1 RNA was 5.2 years (IQR: 2.2–9.9).

**Table 1:** Baseline characteristics of the study population.

Parameter	n = 467
**Demographics**	
**Age** (years); Median (IQR)	49 (45–53)
**Gender**; n (%)	
Male	352 (75.4)
**HIV risk factors**; n (%)	
IDU	226 (48.4)
Heterosexual relations	91 (19.5)
MSM	90 (19.3)
Bisexual relations	7 (1.5)
Other/Unknown	53 (11.3)
**CD4 and HIV viral load**	
**Baseline CD4**; median (IQR)	
CD4 count (cells/μL)	580 (372–781)
CD4%	28 (13)
**Nadir CD4**; median (IQR)	169 (65–274)
**AIDS diagnosis**; n (%)	228 (48.8)
**Time since HIV diagnosis** (years); median (IQR)	17 (10–22)
**Years since undetectable viral load** (<50 copies/mL); median (IQR)	5.2 (2.2–9.9)
**Co-infections**; n (%)	
HBV (HbsAg +)	14 (3.0)
HCV (PCR +)	156 (33.4)
**Reasons for switching**; n (%)	
Drug toxicity/tolerability	197 (42.2)
Physician’s criteria*	133 (28.5)
Unknown reasons	123 (26.3)
Regimen simplification	38 (8.1)
Cost reduction————————————————————————————————————————————————————————————————————————————————————————————————————————————————————————————————————————————————————————————————- Multiple reasons (toxicity-physician’s criteria; physician’s criteria-other, simplification-other)	4 (0.9) 28 (6)
**Previous cART**; n (%)	
***Single-tablet regimen***	
DGV/ABC/3TC	3 (0.6)
RPV/FTC/TDF	4 (0.9)
EFV/FTC/TDF	20 (4.3)
Non-nucleoside reverse transcriptase inhibitor**	
RPV	12 (2.6)
EFV	30 (6.4)
NVP	18 (3.8)
ETV	20 (4.3)
Protease inhibitor**	
LPV	38 (8.1)
FPV	31 (6.6)
ATV unboosted	65 (13.9)
ATV boosted	25 (5.3)
DRV QD	47 (10.1)
DRV BID	19 (4.07)
Integrase strand inhibitor**	
RAL	118 (25.3)
***Nucleoside reverse transcriptase inhibitor***	
ABC/3TC	221 (47.3)
FTC/TDF AZT/3TC/ABC	124 (26.5) 22 (4.7)
3TC	36 (7.7)
ABC	11 (2.3)
FTC	7 (1.5)
TDF	8 (1.7)
AZT ***Entry Inhibitors*** MVC	21 (4.5) 5 (1.1)
**Monotherapy**; n (%)	18 (3.8)
LPV/r	7 (1.5)
DRV/r	11 (2.3)
Dual therapy**; n (%)	17 (3.6)
LPV (+3TC: 3; +RAL: 1) DRV/r (+3TC: 4, +RAL: 3; +ETV: 2; +NVP: 1) ATV/r (+3TC: 1) RAL (+DRV/r: 3; +LPV/r: 1; +ETV: 1; +NVP: 1) Switching to RAL 800 mg QD	4 (0.8) 10 (2.1) 1 (0.2) 6 (1.3) 50 (10.7)

3TC: lamivudine; ABC: abacavir; AIDS: acquired immunodeficiency syndrome; ATV: atazanavir; AZT: zidovudine; cART: combination antiretroviral therapy; DRV: darunavir; DGV: dolutegravir; EFV: efavirenz, ETV: etravirine; HBV: hepatitis B virus; HCV: hepatitis C virus; FPV: fosamprenavir; FTC: emtricitabine; IDU: injection drug use; LPV: lopinavir; MSM: men who have sex with men; MVC: maraviroc; NVP: nevirapine; RPV: rilpivirine; r: ritonavir; TDF: tenofovir; RAL: raltegravir

* Potential drug interactions, better profile, personal decision

**Dual therapy with 3rd agents.

Prior to switching, the treatment regimen included a protease inhibitor (PI) in 225 patients (48.2%), an INSTI in 121 patients (25.9%), a non-nucleoside reverse transcriptase inhibitor (NNRTI) in 104 patients (22.3%), and a regimen with only nucleoside reverse transcriptase inhibitors (NRTI) containing AZT/3TC/ABC in 22 patients (4.7%). Monotherapy with boosted PIs was used in 18 patients and dual therapy in 17 patients including PIs plus 3TC (8), PIs plus INSTI (4), PIs plus NNRTIs (3), and INSTI plus NNRTIs (2). ABC/3TC was the most frequent NRTI backbone co-formulation before switching (221 patients, 47.3%), followed by FTC/TDF (124 patients, 26.5%). In 83 patients (17.8%), the NRTIs were not co-formulated (Table 1).

Data on historical resistance from all previous genotypic studies was available for the 467 patients included in the study. Analyses of these genotypic studies indicated that, before switching, 91 patients had pre-existing resistance mutations; these were to PIs (L10F, V32I, 44D, I50L, and I54V), NNRTI (L100I, K101E, K103N, Y181C, and Y188L), and NRTIs (K65R, K70Q/T, L74I/V, and M184V). Resistance mutation M184I/V had been registered in 23 patients and was detected with L74I/V in 7 patients.

The main reason for switching antiretroviral therapy was drug toxicity/tolerability (197 patients, 42.2%), followed by physician’s criteria (133 patients, 28.5%, which included potential drug interactions, better profile, or personal decision), and other, unknown reasons (123 patients, 26.3%). Among the 197 patients who switched owing to toxicity/tolerability, the specific cause was available in 129 patients, with renal toxicity being the most frequent type (55, 27.9%), followed by bone toxicity (26, 13.2%) and gastrointestinal disorders (22, 11.1%). Most toxicity/tolerability problems were resolved after switching (130, 66%); they remained unresolved in 39 patients (19.8%). No data were available for the other 28 patients.

### Primary endpoint

**Fig 1:**
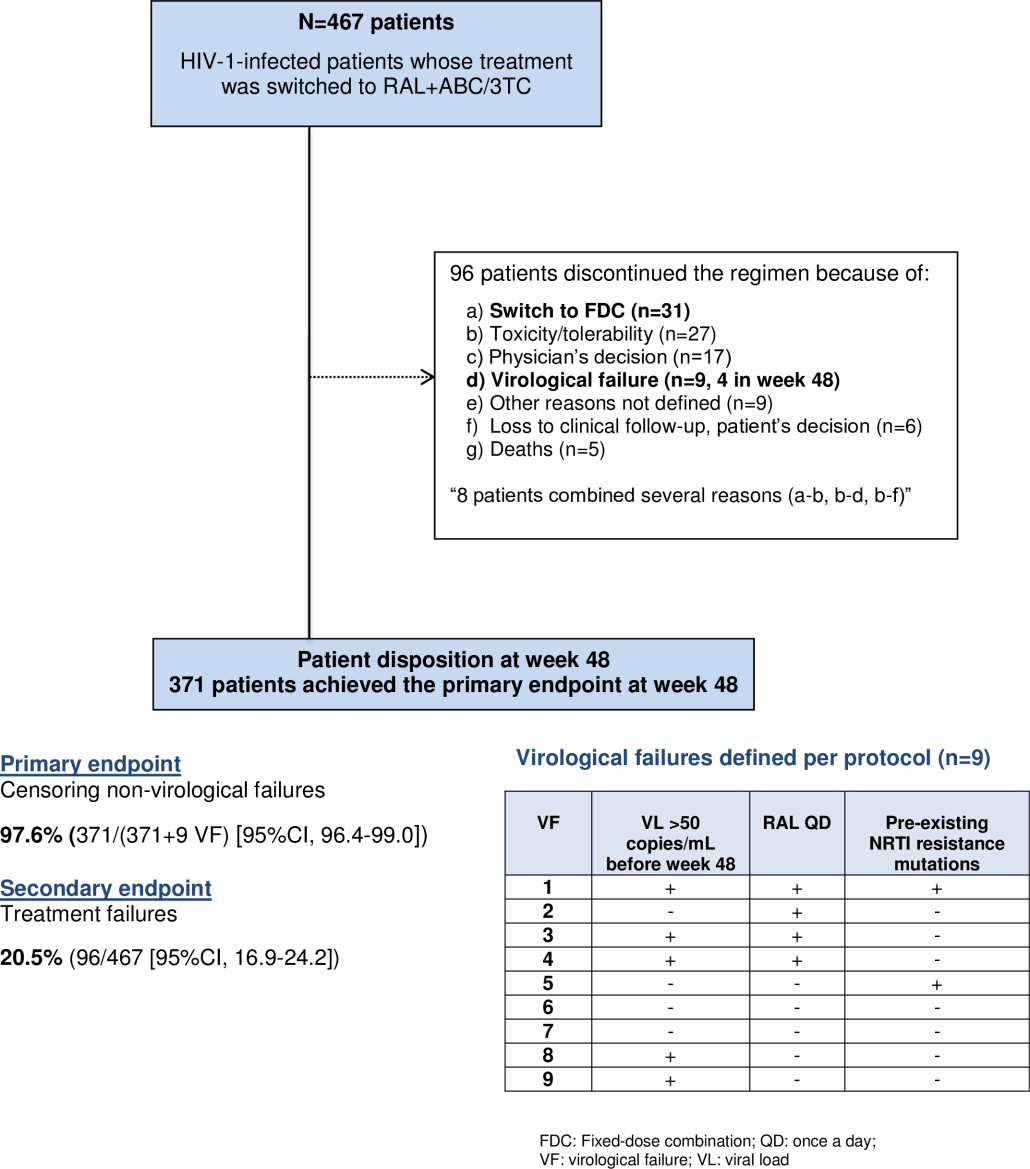
Study flowchart. Of the 9 patients who experienced protocol-defined virological failure, resistance testing was performed in 7 of the 8 patients with a viral load of more than 1,000 copies/m. Mutations compromising RAL, ABC, or 3TC were detected in 6 patients (1.3%). All 3 drugs were involved in 4 patients (0.8%). The mutations included NRTI-resistance mutations (K65R, K70T, L74V, M184V, and T215F) and INSTI resistance mutations (N155H, L163E, and G163H). Previous genotyping showed that 2 of these patients harbored pre-existing NRTI resistance mutations (K65R, K70Q/T, L74I/V, and M184V) before switching to RAL+ABC/3TC. Four of the patients with protocol-defined virological failure were taking RAL once daily following the off-label dosage indication [11], with pre-existing NRTI resistance mutations in 1 of them.

The primary endpoint was achieved by 371/380 patients (97.6% [95% CI, 96.4–99.0]) after censoring non-virological failures, with virological failure in 1.9% of patients. Fig 1.

### Secondary endpoints

#### Treatment failure

**Fig 2:**
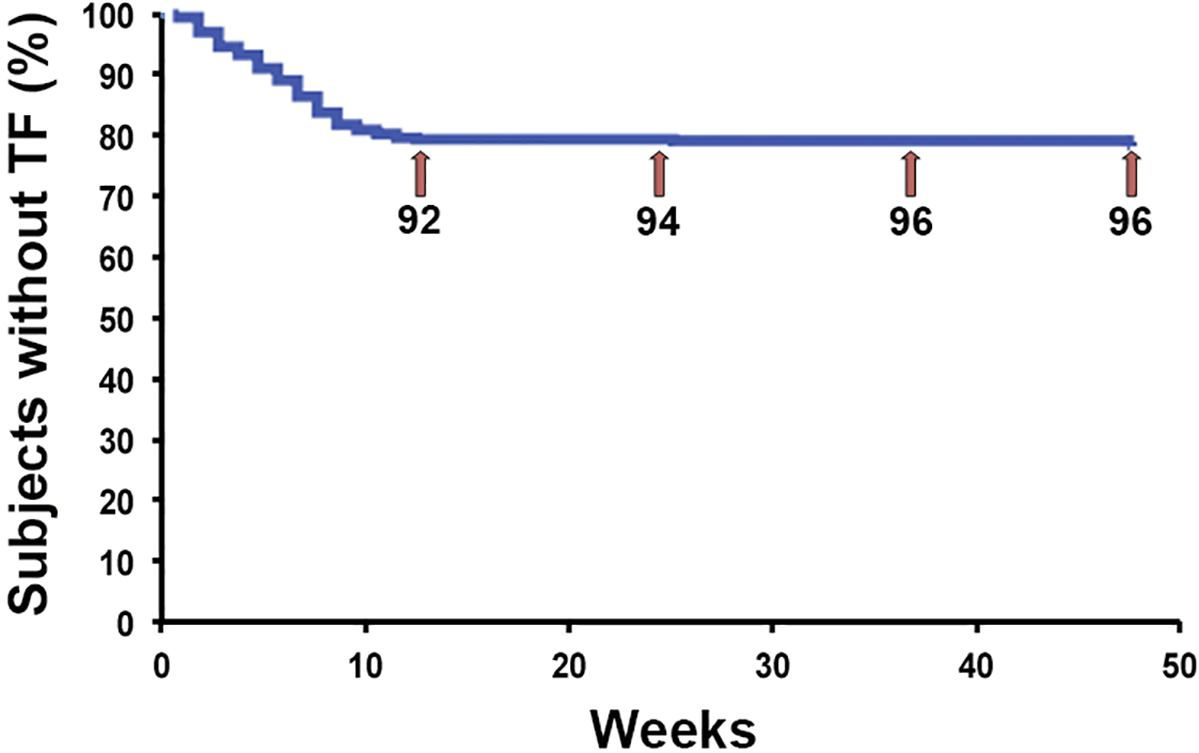
Ad hoc Kaplan-Meier curve. Numbers represent the cumulative number of patients at risk of treatment failure at 12, 24, 36 and 48 weeks.

At 48-week the proportion of patients at risk of treatment failure was 20.5% (96/467 [95%CI, 16.9–24.2]). A survival analysis was performed because more than 10% of failures were for reasons not directly associated with regimen safety or effectiveness. The 48-week overall survival rate was 80% (Fig 2). Of the 467 patients in the study, 92 failed in the first 12 weeks, 2 between 12 and 24 weeks, and 2 between 24 and 36 weeks (Fig 2).

There were 96 failures, and the most frequently reported reasons included switch to fixed-dose combination regimens (31, 6.6%), toxicity/poor tolerability (27, 5.8%), physician’s decision (17, 3.6%), and virological failure (9, 1.9%). Less frequent reasons were loss to clinical follow-up or patient’s decision (6, 1.3%), death not associated with treatment (5, 1.1%), and other (9, 1.9%).

#### Adverse events

Overall, 64 patients (13.7%) experienced 73 AEs (Table 2). The most frequent were systemic (23.3% in 12 patients, 2.6%), digestive (20.5% in 15 patients, 3.2%), and neuropsychiatric (20.3% in 13 patients, 2.7%). AEs resolved in 67.2% of patients (43/64), and medication needed to be changed in 27 patients (5.8%). Clinical intervention was necessary in 20/64 patients (31.2%), and the AE resolved in 15/20 patients (75.0%). In 40/73 events (54.8%), clinicians did not find any relationship between the AE and RAL+ABC/3TC or such a relationship was unlikely. The association with treatment was clear in only 6/73 events (8.2%) and likely related in 27/73 (37.0%). Grade 3 AEs (8 events in 8 patients) were not related to medication, and Grade 2 AEs (14 events in 12 patients) could have been associated with treatment in only 3 patients (25%). Thus, most treatment-associated AEs were Grade 1 (30/33, 90.9%).

**Table 2:** Summary of adverse events.

Trait	n AEs	%AEs	n pts	% pts
**Summary of adverse events**				
Patients with ≥1 AEs			64	13.7
Total number of AEs	73			
Discontinuation due to AEs	29	39.7	27	5.8
Deaths	5	6.8	5	1.1
Resolved	50	68.5	43	67.2
**Types of adverse events**				
Systemic (asthenia, weakness, myalgias, weight loss, anemia, neutropenia.)	17	23.3	12	18.7
Digestive (digestive intolerance, reflux, jaundice, abdominal pain, pancreatitis, hepatic encephalopathy, liver transplant)	15	20.5	15	23.4
Neuropsychiatric (insomnia, depression, anxiety, mood changes)	14	19.1	13	20.3
Dermatological (skin lesions, rash, pruritus)	6	8.2	6	9.4
Infectious (pneumonia, pyelonephritis, *Shigella* ileocolitis, malaria)	5	6.8	5	7.8
Rheumatic (arthralgia) Cancer (lymphoma, duodenal carcinoma, urothelial carcinoma)	4 4	5.5 5.5	4 4	6.2 6.2
Metabolic (hypercholesterolemia, onset of diabetes)	3	4.1	3	4.7
Cardiovascular (myocardial infarction, stroke)	2	2.7	2	3.1
Renal (renal insufficiency)	2	2.7	1	1.6
Respiratory (pulmonary thromboembolism)	1	1.4	1	1.6
**Severity of adverse events**				
Grade 3–4 (fatal or life-threatening)	8	10.9	8	1.7
Grade 2 (requires medical treatment or hospitalization)	14	19.2	12	2.6
Grade 1 (does not require major medical intervention)	51	69.8	44	9.4
**Association of adverse events with RAL+ABC/3TC**				
Related	6	8.2	4	0.9
Likely	27	37	24	5.1
Unlikely	14	19.2	12	2.6
Not related	26	35.6	24	5.1

AEs: adverse event, pts: patients with AEs

#### Changes in blood analytical profiles

**Fig 3:**
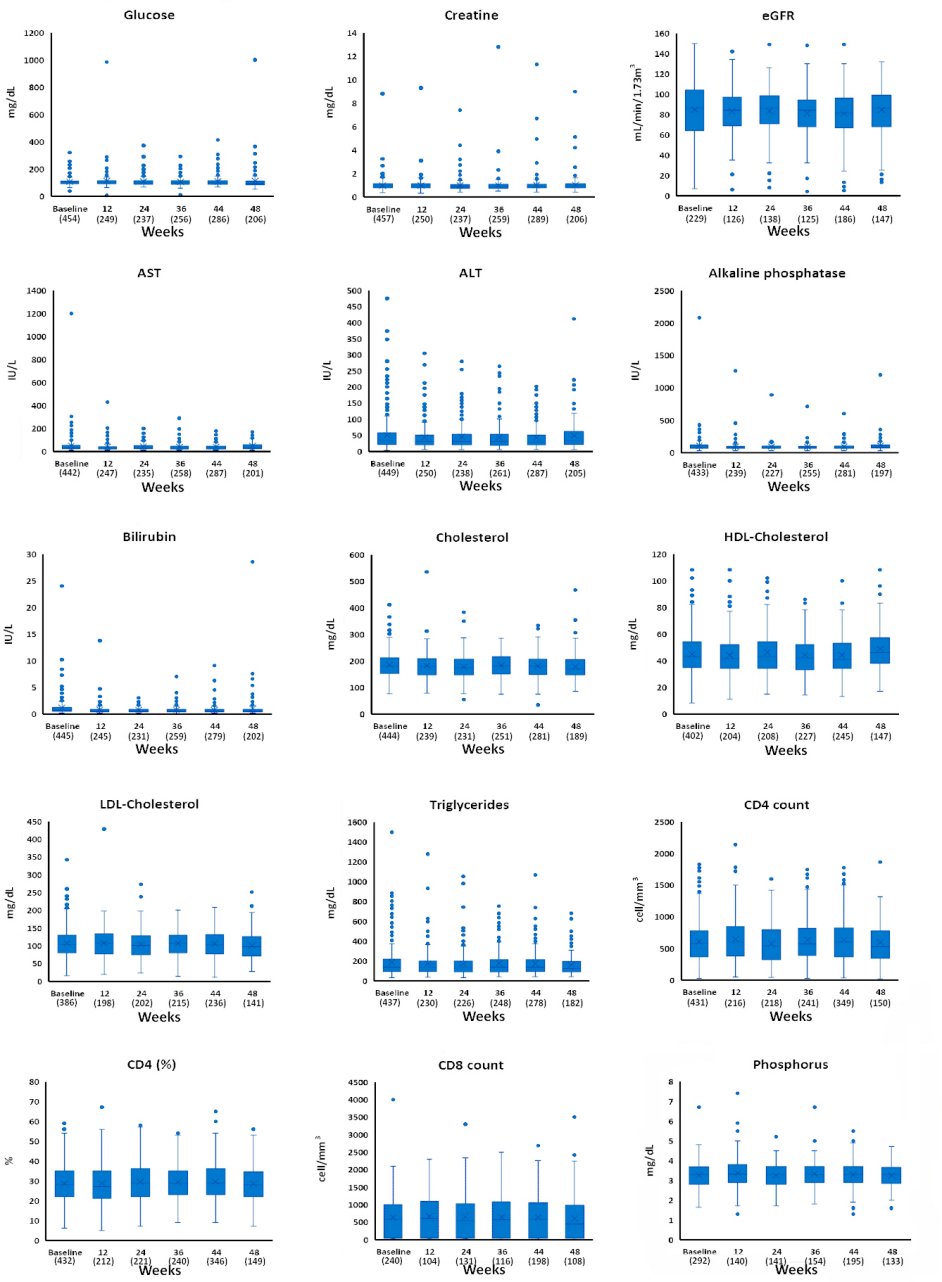
Box-plot representation of lipid, hepatic, and renal profiles from baseline to 48 weeks. Numbers in parenthesis indicate number of patients used to calculate average values at each time point.

Variables related to lipid, hepatic, and renal profiles and CD4+ count (Fig 3) did not change significantly during the 48-week study period (*P*>0.068).

The exceptions were AST, AP, total bilirubin, and HDL cholesterol. HDL cholesterol values were higher at 48 weeks than at any other time (*P*<0.003). More data can be found in Table 3. Similar results were obtained applying GLM analyses (S1 Table), and when the analysis was limited to patients switching from boosted PIs.

**Table 3:** Lipid, hepatic and renal profiles from baseline to 48 weeks.

		n	Median	IQ (25)	IQ (75)	IQR	MLM^1^	LSD^2^
**Glucose**	Baseline	454	98.0	90.0	111.0	21.0		
(mg/dL)	48 weeks	206	96.0	86.0	111.0	25.0		
	Change	201	0.0	-10.0	9.0	19.0	**0.263**	
**Creatinine**	Baseline	457	0.9	0.8	1.1	0.3		
(mg/dL)	48 weeks	206	0.9	0.8	1.1	0.3		
	Change	202	0.0	-0.1	0.1	0.2	**0.900**	
**eGFR**	Baseline	229	86.0	65.0	104.0	39.0		
(mL/min/1.73m^2^)	48 weeks	147	86.0	68.5	99.0	30.5		
	Change	146	0.0	-7.8	6.0	13.7	**0.535**	
**Phosphorus**	Baseline	292	3.3	2.8	3.6	0.8		
(mg/dL)	48 weeks	133	3.3	2.9	3.6	0.7		
	Change	111	0.1	-0.2	0.5	0.7	**0.403**	
**AST**	Baseline	442	31.0	22.0	52.0	30.0		**A**
(IU/L)	48 weeks	201	32.0	23.0	47.0	24.0		**B**
	Change	194	-1.0	-11.0	7.0	18.0	**0.023**	
**ALT**	Baseline	449	33.0	22.0	58.0	36.0		
(IU/L)	48 weeks	205	34.0	22.0	62.0	40.0		
	Change	199	-2.0	-14.5	8.0	22.5	**0.075**	
**Alkaline**	Baseline	433	86.0	67.0	115.0	48.0		**B**
**phosphatase**	48 weeks	197	90.0	73.0	113.0	40.0		**B**
(IU/L)	Change	183	-5.0	-17.5	6.0	23.5	**<10**^**−4**^	
**Bilirubin**	Baseline	445	0.7	0.5	1.3	0.8		**A**
(IU/L)	48 weeks	202	0.6	0.4	0.9	0.5		**A**
	Change	195	-0.1	-0.4	0.1	0.5	**<10**^**−4**^	
**Cholesterol**	Baseline	444	180.0	153.0	212.0	59.0		
(mg/dL)	48 weeks	189	169.0	147.0	204.0	57.0		
	Change	177	-2.0	-26.0	18.0	44.0	**0.360**	
**HDL-cholesterol**	Baseline	402	43.0	35.0	54.0	19.0		**A**
(mg/dL)	48 weeks	147	46.0	38.0	56.5	18.5		**B**
	Change	124	2.0	-4.0	8.2	12.2	**0.001**	
**LDL-cholesterol**	Baseline	386	103.5	80.3	129.7	49.5		
(mg/dL)	48 weeks	141	97.0	72.0	126.0	54.0		
	Change	115	-3.0	-22.0	14.0	36.0	**0.463**	
**Triglycerides**	Baseline	437	137.0	96.0	219.0	123.0		
(mg/dL)	48 weeks	182	123.5	90.0	191.75	101.7		
	Change	165	-10.0	-50.0	27.0	77.0	**0.068**	
**CD4 count**	Baseline	431	580.0	372.0	781.0	409.0		
(cells/mm^3^)	48 weeks	150	529.0	350.8	773.5	422.7		
	Change	137	25.0	-56.0	85.0	141.0	**0.111**	
**CD4 (%)**	Baseline	432	28.0	22.0	35.0	13.0		
	48 weeks	149	28.0	22.0	34.0	12.0		
	Change	137	1.0	-2.0	3.0	5.0	**0.208**	
**CD8 count**	Baseline	240	582.5	57.7	1001.0	943.2		
(cells/mm^3^)	48 weeks	108	456.5	45.7	955.0	909.2		
	Change	100	3.0	-8.2	121.5	129.7	**0.132**	

eGFR: estimated glomerular filtration rate, AST: aspartate aminotransferase, ALT: alanine aminotransferase, GLM: general linear model, LSD: least significant difference.

^1^P-values of mixed linear model analyses comparing variables values at each time point.

^2^Pairwise differences in variable values between time points. A indicates time points with lower values and B intervals with higher values (shown only for variables with significant differences in the MLMs).

Laboratory abnormalities (ie, compared with normal range at baseline) were detected in some patients. These generally did not return to normal when they were due to the effect of previous ARTs or preexisting comorbidities, as reflected in patients with previous renal impairment (Table 4).

**Table 4:** Laboratory abnormalities at baseline before switching and after treatment.

Variable	abnormal at baseline	normal after treatment
Glucose	200	38
Creatinine	93	22
eGFR	126	7
Phosphorus	88	25
AST	1	1
ALT	8	4
AP	58	32
Bilirubin	33	26
Cholesterol	149	47
HDL-cholesterol	94	33
LDL-cholesterol	165	30
Triglycerides	127	36

## Discussion

Although RAL+FTC/TDF has been shown to be highly efficacious and safe as a switching strategy in clinical trials and cohort studies [15,16], equivalent data for RAL+ABC/3TC are scarce, even though this regimen has been used in clinical practice [18]. Therefore, we provide, to our knowledge, data from the largest cohort to date of patients switching to RAL+ABC/3TC based on real-world data.

Our findings revealed a high percentage of treatment success, thus indicating that RAL+ABC/3TC has a high virological suppression rate that is in the same range as other switching therapies [21–22]. Virological failures were reported in only 9 patients (1.9%), and emergent mutations (excluding 2 patients with previous resistance mutations) accounted for 0.8% of the study population. It is important to note that despite the resistance mutations found in historical genotypic studies, that is, M184I/V in 23 patients and simultaneously L74V in 7 patients, only 2 patients experienced virological failure. Given that before switching to RAL+ABC/3TC, most of these patients had been virologically suppressed for more than 2 years, a possible explanation for the low rate of virological failures could be that resistance mutations archived for a long period do not have the same weight as mutations that occurred more recently. Nevertheless, this regimen should not be used in patients with mutations for NRTIs at baseline, because the virological response could be compromised.

Furthermore, 4 of the virological failures were with RAL once daily at the off-label dose, and, as demonstrated in previous studies with naïve patients, this dose could have compromised efficacy [23].

Switching to fixed-dose combination regimens was the main reason for treatment failure, probably because twice-daily dosing of RAL could have hampered adherence to the regimen, especially in recent years, with the increasing availability of new single-tablet options [21, 24,25].

The safety profile of RAL+ABC/3TC is in line with other INI switching studies [21,26], with high overall levels of tolerability, low rates of AEs, and a very low frequency of severe AEs (no Grade 3–4 AEs were related to treatment). Neuropsychiatric AEs have recently been associated with INSTIs as a family. In our cohort, insomnia, depression, anxiety, and mood changes appeared in only 2.7% of patients. These AEs were mild in 85.6% of cases and were not clearly related to treatment in 71.4% of cases. These findings are better than those for the INSTI dolutegravir, with a slightly higher rate of neuropsychiatric AEs and discontinuations due to these symptoms [27].

It is also important to note that most toxicity/tolerability problems related to previous regimens were resolved after the switch to RAL+ABC/3TC.

No significant changes in lipids were observed, including in the subgroup of patients who had previously taken boosted PI–based regimens, although significant changes in lipid values have been reported in other cohorts. These findings could have 2 explanations. On the one hand, most of the patients who switched from PIs (69%) were taking darunavir or atazanavir. These drugs have a much more neutral lipid profile than lopinavir or fosamprenavir, which are more common in previous cohort studies. On the other hand, 25% of patients were already taking RAL and 50% were taking ABC/3TC[15, 16].

Renal function parameters also remained stable during the study period, even in the subgroup of patients who switched from a regimen containing TDF (*P*<0.975). These findings differ from those of other studies on switching from TDF to ABC, which reported a slight increase in eGFR [28, 29]. A possible explanation could be that around 50% of patients were on ABC/3TC before switching, and TDF-containing regimens only accounted for 26% of patients.

In addition, although we did not document a significant increase in the CD4+ lymphocyte count, it should be noted that the baseline median CD4+ lymphocyte count was over 500 cells/mm^3^, which is in the normal range for HIV-1-negative patients. Moreover, some patients had an undetectable viral load for more than 5 years prior to switching.

Our study is subject to limitations. First, it was a retrospective study with no control group. Second, clinical protocols and visit timetables differed between the participating hospitals. Nevertheless, to our knowledge, we report the largest series to date, with 467 patients receiving RAL+ABC/3TC as a switching strategy. Based on these good results, this combination could potentially form the basis for a switching strategy against the toxicity of TDF, when tenofovir alafenamide is not available [30] or for other clinical reasons.

The availability of a generic co-formulation of ABC/3TC with a more competitive price makes this regimen a cheaper switching strategy than the most common antiretroviral regimens that include boosted PIs and other integrase inhibitors [31]. This could have a significant economic impact on hospital drug expenditure [32].

A new formulation of RAL, 1200 mg QD, is now available for naïve patients and will make dosing easier [33] and improve adherence. Nevertheless, this strategy should be evaluated carefully, taking into account the virological failures reported with RAL 800 mg QD, and lack of efficacy due to historical genotypic mutations that can be archived from previous treatments.

In summary, the results of the KIRAL study show that RAL+ABC/3TC is an effective, safe, well-tolerated, and inexpensive switching strategy in patients with virologically stable HIV-1 infection.

## Supporting information

S1 Table Lipid, hepatic and renal profiles from baseline to 48 weeks (GLM analysis). (DOCX)
