# Supplementary material for: Raltegravir plus abacavir/lamivudine in virologically suppressed HIV-1-infected patients: 48-week results of the KIRAL study
Source: PLoS One. 2018 Jun 14;13(6):e0198768. doi: 10.1371/journal.pone.0198768 (PMC6002106; doi:10.1371/journal.pone.0198768)
Supplement: S1 Table — (DOCX) [file pone.0198768.s001.docx]

Lipid, hepatic and renal profiles from baseline to 48 weeks (GLM analysis)

|  |  | **n** | **Median** | **IQ (25)** | **IQ (75)** | **IQR** | **GLM^1^** | **LSD^2^** |
| --- | --- | --- | --- | --- | --- | --- | --- | --- |
| **Glucose** | Baseline | 454 | 98.0 | 90.0 | 111.0 | 21.0 |  |  |
| (mg/dL) | 48 weeks | 206 | 96.0 | 86.0 | 111.0 | 25.0 |  |  |
|  | Change | 201 | 0.0 | -10.0 | 9.0 | 19.0 | **0.633** |  |
| **Creatinine** | Baseline | 457 | 0.9 | 0.8 | 1.1 | 0.3 |  |  |
| (mg/dL) | 48 weeks | 206 | 0.9 | 0.8 | 1.1 | 0.3 |  |  |
|  | Change | 202 | 0.0 | -0.1 | 0.1 | 0.2 | **0.907** |  |
| **eGFR** | Baseline | 229 | 86.0 | 65.0 | 104.0 | 39.0 |  |  |
| (mL/min/1.73m^2^) | 48 weeks | 147 | 86.0 | 68.5 | 99.0 | 30.5 |  |  |
|  | Change | 146 | 0.0 | -7.8 | 6.0 | 13.7 | **0.534** |  |
| **Phosphorus** | Baseline | 292 | 3.3 | 2.8 | 3.6 | 0.8 |  |  |
| (mg/dL) | 48 weeks | 133 | 3.3 | 2.9 | 3.6 | 0.7 |  |  |
|  | Change | 111 | 0.1 | -0.2 | 0.5 | 0.7 | **0.510** |  |
| **AST** | Baseline | 442 | 31.0 | 22.0 | 52.0 | 30.0 |  | **A** |
| (IU/L) | 12 weeks | 247 | 38.0 | 21.0 | 41.0 | 20.0 |  | **B** |
|  | 24 weeks | 235 | 37.0 | 22.0 | 49.5 | 27.5 |  | **B** |
|  | 36 weeks | 258 | 35.0 | 21.0 | 46.0 | 25.0 |  | **B** |
|  | 44 weeks | 287 | 35.0 | 21.0 | 45.0 | 24.0 |  | **B** |
|  | 48 weeks | 201 | 32.0 | 23.0 | 47.0 | 24.0 |  | **AB** |
|  | Change | 194 | -1.0 | -11.0 | 7.0 | 18.0 | **0.046** |  |
| **ALT** | Baseline | 449 | 33.0 | 22.0 | 58.0 | 36.0 |  |  |
| (IU/L) | 48 weeks | 205 | 34.0 | 22.0 | 62.0 | 40.0 |  |  |
|  | Change | 199 | -2.0 | -14.5 | 8.0 | 22.5 | **0.131** |  |
| **Alkaline** | Baseline | 433 | 86.0 | 67.0 | 115.0 | 48.0 |  | **B** |
| **phosphatase** | 12 weeks | 239 | 77.0 | 62.0 | 97.0 | 35.0 |  | **A** |
| (IU/L) | 24 weeks | 227 | 82.0 | 65.0 | 102.5 | 37.5 |  | **A** |
|  | 36 weeks | 255 | 77.0 | 62.0 | 96.5 | 34.5 |  | **A** |
|  | 44 weeks | 281 | 81.0 | 64.0 | 100.0 | 36.0 |  | **A** |
|  | 48 weeks | 197 | 90.0 | 73.0 | 113.0 | 40.0 |  | **B** |
|  | Change | 183 | -5.0 | -17.5 | 6.0 | 23.5 | **0.005** |  |
| **Bilirubin** | Baseline | 445 | 0.7 | 0.5 | 1.3 | 0.8 |  | **A** |
| (IU/L) | 12 weeks | 245 | 1.0 | 1.0 | 2.5 | 1.5 |  | **B** |
|  | 24 weeks | 231 | 2.0 | 1.5 | 2.5 | 1.0 |  | **B** |
|  | 36 weeks | 259 | 4.0 | 3.5 | 5.5 | 2.0 |  | **B** |
|  | 44 weeks | 279 | 1.0 | 1.0 | 2.5 | 1.5 |  | **B** |
|  | 48 weeks | 202 | 0.6 | 0.4 | 0.9 | 0.5 |  | **A** |
|  | Change | 195 | -0.1 | -0.4 | 0.1 | 0.5 | **<10^-4^** |  |
| **Cholesterol** | Baseline | 444 | 180.0 | 153. | 212.0 | 59.0 |  |  |
| (mg/dL) | 48 weeks | 189 | 169.0 | 147. | 204.0 | 57.0 |  |  |
|  | Change | 177 | -2.0 | -26.0 | 18.0 | 44.0 | **0.513** |  |
| **HDL-cholesterol** | Baseline | 402 | 43.0 | 35.0 | 54.0 | 19.0 |  | **A** |
| (mg/dL) | 12 weeks | 204 | 41.5 | 34.0 | 52.0 | 18.0 |  | **A** |
|  | 24 weeks | 208 | 43.0 | 34.0 | 54.0 | 20.0 |  | **A** |
|  | 36 weeks | 227 | 42.0 | 33.5 | 52.0 | 18.5 |  | **A** |
|  | 44 weeks | 245 | 41.0 | 34.0 | 53.0 | 19.0 |  | **A** |
|  | 48 weeks | 147 | 46.0 | 38.0 | 56.5 | 18.5 |  | **B** |
|  | Change | 124 | 2.0 | -4.0 | 8.2 | 12.2 | **0.031** |  |
| **LDL-cholesterol** | Baseline | 386 | 103.5 | 80.3 | 129.7 | 49.5 |  |  |
| (mg/dL) | 48 weeks | 141 | 97.0 | 72.0 | 126.0 | 54.0 |  |  |
|  | Change | 115 | -3.0 | -22.0 | 14.0 | 36.0 | **0.533** |  |
| **Triglycerides** | Baseline | 437 | 137.0 | 96.0 | 219.0 | 123.0 |  |  |
| (mg/dL) | 48 weeks | 182 | 123.5 | 90.0 | 191.75 | 101.7 |  |  |
|  | Change | 165 | -10.0 | -50.0 | 27.0 | 77.0 | **0.404** |  |
| **CD4 count** | Baseline | 431 | 580.0 | 372.0 | 781.0 | 409.0 |  |  |
| (cells/mm^3^) | 48 weeks | 150 | 529.0 | 350.8 | 773.5 | 422.7 |  |  |
|  | Change | 137 | 25.0 | -56.0 | 85.0 | 141.0 | **0.172** |  |
| **CD4 (%)** | Baseline | 432 | 28.0 | 22.0 | 35.0 | 13.0 |  |  |
|  | 48 weeks | 149 | 28.0 | 22.0 | 34.0 | 12.0 |  |  |
|  | Change | 137 | 1.0 | -2.0 | 3.0 | 5.0 | **0.626** |  |
| **CD8 count** | Baseline | 240 | 582.5 | 57.7 | 1001.0 | 943.2 |  |  |
| (cells/mm^3^) | 48 weeks | 108 | 456.5 | 45.7 | 955.0 | 909.2 |  |  |
|  | Change | 100 | 3.0 | -8.2 | 121.5 | 129.7 | **0.977** |  |

^eGFR: estimated glomerular filtration rate, AST: aspartate aminotransferase, ALT: alanine aminotransferase, GLM: general linear model, LSD: least significant difference.^

^1^P-values of GLM analyses comparing variables values at each time point.

^2^Pairwise differences in variable values between time points. A indicates time points with lower values and B intervals with higher values (shown only for variables with significant differences in the GLMs).
